# Premature Ventricular Complex-Induced Cardiomyopathy, a Review: Current Insights, Diagnostic Challenges, and Therapeutic Strategies

**DOI:** 10.3390/jcm15041360

**Published:** 2026-02-09

**Authors:** Mario J. Recio-Ibarz, Teresa Olóriz, Naiara Calvo, Beatriz Jáuregui, Vanesa Alonso-Ventura, Daniel Cantero Lozano, Carlos López-Perales

**Affiliations:** 1 Arrhythmia Unit, Cardiology Department, Miguel Servet University Hospital, Paseo de Isabel la Católica 1-3, 50009 Zaragoza, Spain; merecio976@gmail.com (M.J.R.-I.);; 2 Department of Medicine, Psychiatry and Dermatology, University of Zaragoza, 50009 Zaragoza, Spain

**Keywords:** premature ventricular complexes, PVC-induced cardiomyopathy, catheter ablation, antiarrhythmic therapy, heart failure, electrocardiographic imaging, artificial intelligence

## Abstract

Premature Ventricular Complexes (PVCs) are among the most frequent ventricular arrhythmias observed in daily cardiology practice. Although often benign, sustained high ectopic activity can result in left ventricular dysfunction known as PVC-induced Cardiomyopathy (PVC-CMP), a condition that is frequently reversible when the arrhythmia is effectively suppressed. The underlying mechanisms are multifaceted, involving electromechanical dyssynchrony, contractile inefficiency, abnormal calcium cycling, neurohormonal activation, and progressive structural remodeling. The likelihood of developing PVC-CMP varies among individuals and is influenced by electrophysiological and structural factors. Diagnosis relies on prolonged rhythm monitoring, comprehensive multimodality imaging, and demonstration of ventricular recovery after reducing the ectopic burden, which, in turn, confirms causality. Over the past decade, major advances in electrocardiographic mapping, cardiac imaging, and ablation therapy have transformed this field, demonstrating excellent efficacy and safety profiles. In parallel, artificial intelligence and computational mapping are emerging as powerful tools for prediction and procedural guidance. Recognition of PVC-CMP as a distinct, treatable cardiomyopathy highlights the importance of early detection and individualized therapy, offering the prospect of complete functional recovery and the prevention of heart failure progression.

## 1. Introduction

Premature ventricular complexes (PVCs) are a frequent finding in routine practice and may occur across the full spectrum of health and disease. A standard 12-lead electrocardiogram (ECG) can reveal PVCs in roughly 1–4% of unselected adults, while prolonged ambulatory recordings show ectopy in most individuals, particularly with advancing age or comorbidity [1,2].

Historically regarded as harmless in structurally normal hearts, frequent monomorphic PVCs were later linked to reversible left ventricular (LV) function impairment. Early case reports demonstrated restoration of LV systolic function once the ectopy was suppressed [3,4,5]. Subsequent systematic investigations and registry data, together with contemporary state-of-the-art reviews that broadened the conceptual framework of arrhythmia-induced cardiomyopathy, refined the understanding of which patients may be vulnerable, what arrhythmic or host factors may predict non-recovery, and how medical therapy, ablation, or hybrid approaches behave in controlling ectopy and restoring LV function [6,7,8]. Moreover, advances in mapping technologies, non-invasive electrocardiographic imaging (ECGi), and machine-learning-based analysis have increased diagnostic precision and procedural efficiency for PVC localization and ablation [9,10,11,12].

## 2. Methods

This manuscript is a narrative, non-systematic review focused on PVC-induced Cardiomyopathy (PVC-CMP). A structured literature search was conducted from January 2000 to October 2025 across PubMed/MEDLINE and Embase, with complementary searches in the Cochrane Library and manual screening of reference lists from relevant guidelines, consensus statements, and high-impact reviews.

Search strategies combined free-text keywords and controlled vocabulary terms, including but not limited to PVCs, PVC-CMP, ventricular ectopy, arrhythmia-induced cardiomyopathy, catheter ablation, electrophysiological mapping, cardiac imaging, and artificial intelligence (AI), using Boolean operators.

Eligible publications included European Society of Cardiology (ESC) guidelines and society documents, systematic reviews and meta-analyses, randomized and non-randomized trials, prospective and retrospective cohorts, multicenter registries, and selected translational studies relevant to PVC-CMP mechanisms and imaging. Exclusion criteria included non-English articles, abstracts without full text, single case reports (unless historically seminal), and studies not specifically addressing PVCs or PVC-CMP.

Article screening and selection were performed independently by the authors based on relevance to the predefined review objectives. Overall, approximately 900 records were identified through database searching and complementary sources; approximately 130 full-text articles were assessed for eligibility, and 42 publications were retained for qualitative synthesis. Given the narrative nature of the review, no formal systematic protocol, risk-of-bias assessment, or quantitative synthesis was applied.

## 3. Epidemiology, Clinical Profiles, and Phenotypic Spectrum of PVCs

### 3.1. Prevalence and Population Differences

Estimates of PVC prevalence vary widely because detection increases with longer monitoring and higher recording resolution. Reviews and guideline documents report that extended Holter or event monitoring can identify PVCs in 40–75% of individuals, with reported burdens influenced by monitoring duration, referral patterns, and comorbidity profiles [1,2,13,14].

PVC frequency rises with age, is slightly higher in men, and correlates with conventional cardiovascular risk factors [1,13]. Large population studies link higher PVC frequency to taller stature, elevated systolic pressure, smoking, structural heart disease, coronary atherosclerosis, sleep disordered breathing, and systemic inflammation [2,13]. Diabetes and chronic kidney disease are also associated with more frequent PVCs and poorer outcomes, mainly through pathways involving diffuse fibrosis, autonomic imbalance, and metabolic stress [1,2].

Long-term follow-up indicates that spontaneous improvement of idiopathic PVCs is more common than previously assumed. In an untreated cohort with preserved ventricular function, a substantial proportion (44%) of patients showed meaningful reductions in burden (<1%) over time, while the remaining patients showed stable PVC burden [1,2]. In addition, in this study, the baseline burden did not influence the chance of PVCs reduction during follow-up. This pattern of improvement appears particularly evident in idiopathic PVCs originating from right-sided chambers and outflow tract (OT) regions, a phenotype repeatedly described as benign and more prone to variability in the absence of structural disease or cardiovascular comorbidity. Conversely, PVCs with LV, epicardial, or intramural origins tend to show less spontaneous burden reduction during follow-up [1,2,14,15].

Furthermore, comorbidities not only predispose to ectopy but also shape its clinical impact. Depending on myocardial reserve, even modest ectopic loads may impair ventricular function, whereas younger individuals with structurally normal hearts often tolerate higher burdens before ventricular decline appears [6,16].

### 3.2. Anatomical Origins, Mechanisms, and Relation to Underlying Cardiomyopathy

**Figure 1:**
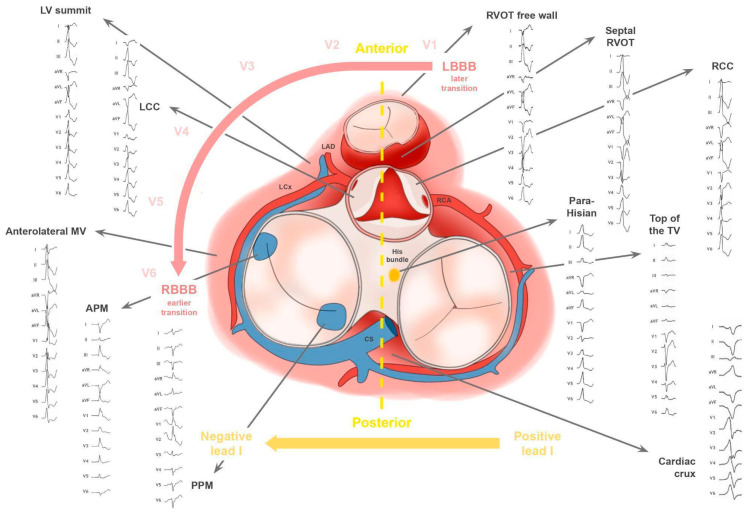
Schematic electrocardiographic representation of the main foci of idiopathic PVCs (adapted from Ref. [20]).

PVCs usually arise from a limited number of myocardial regions (Figure 1), often located near junctional zones or peri-annular areas where myocardial fibers intermix with elements of the conduction system. This anatomical arrangement predisposes these areas to focal automaticity or triggered activity. The ventricular OTs, particularly the right ventricular outflow tract (RVOT), represent the most common source of idiopathic monomorphic PVCs, accounting for approximately 60–80% of idiopathic cases and roughly 20–40% of ablation procedures [10,17,18]. Other less frequent origins include the left ventricular outflow tract (LVOT) (about 10–20%), fascicular regions (5–10%), and annular, papillary muscle, moderator band, or LV summit and crux sites, each representing <10% of idiopathic presentations [1,9,10,17,18,19,20].

Distribution patterns and clinical implications vary with both patient profile and arrhythmic substrate. OT and fascicular PVCs are typically benign and rarely associated with sustained ventricular arrhythmias. In contrast, Purkinje-related, scar-associated, papillary muscle, and epicardial or intramural foci carry higher arrhythmic and prognostic risk [9,10,20,21]. Recent population-based data further support the prognostic relevance of PVC location, showing that LV intramyocardial and epicardial origins independently predict incident heart failure (HF), even after accounting for PVC burden and conventional cardiovascular risk factors [18]. Recognizing the anatomic origin and associated risk profile is therefore essential to guide monitoring, prognostic assessment, and management strategies.

OT PVCs are typically mediated by cyclic AMP-dependent triggered activity via delayed afterdepolarizations and are thus both adenosine-sensitive and catecholamine-responsive, explaining their circadian variability and partial response to β-blockade [10,19]. These arrhythmias are usually benign and occur in otherwise healthy individuals, and their burden can fluctuate over time in response to autonomic tone, emotional stress, stimulant intake, or hormonal changes. In many cases, especially among younger patients without structural heart disease, spontaneous remission or long-term reduction in ectopic activity may occur [1,10,19].

Fascicular PVCs arise from the LV conduction system, most commonly along the posterior or anterior fascicles of the left bundle branch. They are characteristically mediated by a verapamil-sensitive reentrant mechanism, producing relatively narrow QRS complexes and short coupling intervals. Fascicular PVCs are generally benign and drug-responsive, but may occasionally trigger fascicular ventricular tachycardia, particularly in younger individuals [9,10,20].

Annular PVCs, which are less frequent, originate from the mitral or tricuspid annuli through mechanisms similar to OT forms. They often mimic RVOT or LVOT morphologies on surface ECG but require distinct mapping and ablation strategies, targeting the atrioventricular junctions or the coronary venous system [9,10].

Purkinje-related PVCs constitute a distinct and clinically relevant entity, arising from regions with enhanced automaticity or microreentrant activity within the Purkinje network. These arrhythmias are notable for their propensity to trigger malignant ventricular events, making careful evaluation and individualized management essential. Likewise, moderator band and right ventricular septal PVCs, in which Purkinje fibers interdigitate with the trabeculated myocardium, can also act as triggers for polymorphic ventricular arrhythmias, particularly in post-infarction hearts or in the presence of structural disease [10,21,22].

Scar-associated PVCs originate from micro- or macroreentrant circuits within fibrotic or infarct border zones, characterized by slow, fractionated conduction. They often reflect underlying structural disease and can trigger or sustain ventricular tachycardia or fibrillation, conferring higher arrhythmic and prognostic risk. These PVCs are generally less amenable to focal ablation and may require broader substrate modification or multimodal mapping approaches for effective control [10,21,22,23].

Papillary muscle PVCs arise from small, mechanically stressed myocardial bundles, most often the posteromedial or anterolateral LV papillary muscles. Their complex motion and their intramural or epicardial extensions create challenges during ablation, lead to unstable catheter contact, and contribute to high recurrence rates. These foci may exhibit multiple or shifting exit sites and, in some predisposed patients, can trigger malignant ventricular arrhythmias [10,21].

Finally, LV summit and crux PVCs represent some of the most challenging idiopathic subtypes due to their epicardial or intramural origin and their proximity to major coronary vasculature [10,19,21]. The LV summit region, situated between the left anterior descending and left circumflex arteries, is frequently inaccessible via standard endocardial routes and often necessitates coronary venous, epicardial, or hybrid ablation approaches [10]. In addition, crux PVCs, which arise near the posteroseptal intersection of the atrioventricular grooves, lie in proximity to key vascular structures, including the posterior descending artery, the atrioventricular nodal artery, and adjacent coronary venous branches. This anatomic relationship can contribute to broad breakout patterns and variable preferential conduction, often requiring alternative advanced mapping strategies to achieve successful ablation [19,21]. These characteristics make LV summit and crux PVCs especially resistant to conventional ablation and increase the likelihood of recurrence [10].

### 3.3. Symptoms, Hemodynamic Effects, and Quality of Life

Symptoms range from isolated palpitations to profound fatigue, exercise intolerance, or HF manifestations and do not correlate strictly with PVC burden. Even low ectopic burdens can be distressing in sensitive patients, whereas others may remain asymptomatic despite a high burden [1,2]. Quality-of-life impairment is well documented; symptom resolution after effective therapy correlates with improvements in fatigue, exercise tolerance, and patient-reported well-being, whereas persistent ectopy is associated with anxiety and reduced activity levels [8,24,25]. Most importantly, PVCs can also lead to PVC-CMP with progressive remodeling if untreated, contributing to significant morbidity and HF progression [6,7,15,25].

## 4. Pathophysiology, Genetics, and Risk of PVC-CMP

### 4.1. Mechanistic Pathways from Repetitive Ectopy to Cardiomyopathy

PVC-CMP represents an acquired yet often reversible form of LV dysfunction in which chronic ectopic activation leads to mechanical inefficiency and maladaptive remodeling. Importantly, a spectrum exists between true PVC-CMP (where ectopy is the primary driver) and PVC-aggravated cardiomyopathy (where frequent PVCs worsen an underlying or subclinical myocardial disease), and the distinction is often inferred from the degree and time course of LV recovery after effective PVC suppression [6,7]. The principal mechanism is electromechanical dyssynchrony, whereby repetitive premature depolarizations induce regional contraction discoordination, altered wall stress, and reduced stroke work. Early investigations proposed that both inter- and intraventricular dyssynchrony, as well as loss of coordinated torsion, contribute to this process [3,6,7]. Experimental data further implicate abnormal calcium handling, mitochondrial dysfunction, oxidative stress, and interstitial fibrosis as secondary contributors to contractile impairment. However, these alterations are not unique to PVC-CMP and may overlap with mechanisms observed in other cardiomyopathies, particularly in PVC-aggravated phenotypes [6,7,16].

The duration of exposure to frequent PVCs has been established as a major determinant of cardiomyopathy development. Symptom duration of 30–60 months confers a fourfold higher risk of PVC-CMP, and over 60 months up to a twentyfold increase, while asymptomatic patients are particularly vulnerable owing to prolonged unrecognized exposure [6,15,16].

Electrical and anatomical characteristics further shape risk. A PVC-QRS width exceeding 150 ms identifies patients at increased risk of LV dysfunction, supporting the role of electrical and mechanical dyssynchrony. LV locations, particularly epicardial, intramural, or multifocal PVC, have been associated with higher long-term event rates in population cohorts, supporting a more nuanced interpretation when burden alone does not fully explain clinical findings [6,10]. This helps refine attribution in borderline cases where causality is uncertain, underscoring the pivotal role of activation patterns and timing in disease development. Additional factors include the absence of circadian variation and interpolated PVCs: a uniform hourly distribution predicts LV dysfunction regardless of total burden, while interpolation increases effective exposure by allowing more ectopy within similar heart rates [6,16].

Hemodynamically, repetitive PVCs intermittently reduce preload, leading to fluctuations in stroke volume. When the burden is high, mean cardiac output declines, triggering neurohormonal activation, including elevations in natriuretic peptides and stimulation of the renin–angiotensin–aldosterone system, which, in turn, accelerates LV dilation and adverse structural remodeling [6,7]. The extent of myocardial fibrosis ultimately determines the degree of functional recovery once ectopy is suppressed. Restoration of LV function after successful medical or ablation therapy confirms both causality and the potential for reversibility in the absence of irreversible scarring [3,25].

### 4.2. Genetic Predisposition and Modifiers of Susceptibility

Genetic variation influences individual tolerance and response to frequent ectopy [1]. Recent genotype-phenotype studies have identified variants in desmosomal genes (DSP, PKP2), structural or cytoskeletal proteins (LMNA, TTN, DES), and ion-handling regulators (SCN5A, RYR2, PLN) that increase susceptibility to arrhythmia-related LV dysfunction and may unmask subclinical cardiomyopathic substrates. These variants are also linked to concealed cardiomyopathic phenotypes, suggesting that in some patients, PVCs may reflect an underlying genetic predisposition rather than a truly idiopathic origin. These genetic forms often exhibit variable penetrance and incomplete expression, which may explain why overt cardiomyopathy develops only in a subset of carriers [24,25].

Shared molecular pathways involving desmosomal remodeling and ion-channel dysregulation have also been described, linking PVC-CMP with early arrhythmogenic or dilated cardiomyopathic phenotypes. Genetic predisposition may lower the threshold of ectopic burden required to induce LV dysfunction, accounting for interindividual variability in disease expression. Recognition of these genetic markers has important implications for family screening and for determining when earlier intervention should be considered in young or borderline cases, as even moderate PVC burdens can be deleterious in genetically predisposed hearts [1,24,25].

### 4.3. Burden-Dependent Risk and Pitfalls in Attribution

Multiple observational cohorts and case–control studies have examined the relationship between PVC burden and LV function [6,15,16]. Although no absolute threshold exists, burdens above approximately 10–20% are commonly associated with measurable functional impairment, and several series suggest a burden threshold around 20–24%, beyond which LVEF is more likely to be reduced [15,16]. In this range, persistent high-burden ectopy is a well-established risk factor for the development of tachycardiomyopathy and therefore warrants definitive therapy or close longitudinal follow-up [6,9]. However, clinically significant PVC-CMP can also occur at lower burdens (even 5–10%) in susceptible individuals, underscoring not only interindividual variability but also the presence of underlying structural heart disease as a key determinant of vulnerability [6,7]. Accordingly, in patients with low PVC burden and LV dysfunction, a causal relationship should be cautiously considered and supported by additional features such as younger age, absence of late gadolinium enhancement (LGE) on CMR, or documented recovery after PVC suppression [16,25]. In this setting, premature attribution of systolic dysfunction to PVCs may delay recognition of an ischemic or primary cardiomyopathy [6,16].

### 4.4. Impact on Cardiac Resynchronization (CRT) and Pacing Therapies

In patients receiving CRT, frequent PVCs can markedly reduce the proportion of effective biventricular pacing, thereby diminishing hemodynamic benefit and limiting reverse remodeling. This phenomenon reflects the same pathophysiological mechanisms underlying PVC-CMP, including mechanical inefficiency and ventricular dyssynchrony [6,7,9]. Even modest reductions in biventricular pacing due to PVC interference have been associated with worse clinical outcomes and higher rates of CRT non-response in device-based and CRT-specific cohorts [26,27]. Dedicated rhythm assessment (e.g., Holter monitoring), is therefore essential to accurately quantify ectopic burden and identify loss of effective pacing not captured by device counters [1,14,28]. Importantly, ablation or effective medical suppression of PVCs has been shown to restore effective biventricular pacing and improve functional response to CRT [25,26]. Consequently, in CRT recipients with a substantial PVC burden, systematic evaluation and targeted ectopy suppression are strongly recommended to optimize biventricular pacing and maximize clinical benefit, in accordance with current ESC guidance [9].

## 5. Diagnostic Approach: Quantification, Imaging, and Localization

The following subsections summarize evidence-based strategies, addressing each of the three fundamental questions regarding effective PVC-CMP management:

1. Is the PVC burden sufficient to cause cardiomyopathy?
2. Is there any underlying structural condition contributing to the ectopy?
3. Where is the PVC site of origin, and can it be targeted for ablation?

### 5.1. Ambulatory Monitoring and PVC Burden Quantification

Accurate assessment of PVC frequency requires extended rhythm monitoring or, at least, serial Holter monitoring, as day-to-day variability is common and short recordings tend to underestimate ectopy [1,14]. Consensus documents and large cohort studies recommend at least 24 to 48 h of continuous Holter monitoring for initial evaluation, extending up to seven days or to implantable loop recording when intermittent high burden is suspected [9,14]. This strategy provides the most reliable quantification in borderline or fluctuating cases [1,9].

Data from pacemakers or implantable cardioverter-defibrillators can also provide continuous ectopic counts and long-term trends, supporting clinical decision-making and longitudinal follow-up [27,28]. Consumer-grade wearables and single-lead ECG devices have advanced rapidly, and validation studies demonstrate reasonable accuracy for PVC detection when algorithms are appropriately trained. However, they remain less reliable than conventional ambulatory systems [28,29].

### 5.2. Imaging: Echocardiography and CMR, Prognostic Implications, and Other Imaging Techniques

Transthoracic echocardiography (TTE) remains the primary imaging modality for evaluating the impact of PVCs on cardiac structure and function. TTE provides real-time assessment and avoids artifacts that may occur with non-real-time modalities such as CMR [7]. During TTE, the temporal correlation between patient-reported symptoms and PVC occurrence can sometimes be assessed; however, this correlation is often modest, highlighting the limited reliability of symptom-ectopy concordance during imaging acquisition [1]. Of note, in patients with frequent PVCs, LVEF assessment should ideally be performed on the sinus beat following the first post-extrasystolic contraction, or reported as an averaged value across multiple beats when this is not feasible (e.g., bigeminal rhythms) [7,30]. Beyond structural evaluation, TTE also provides prognostic information: subtle abnormalities, such as reduced global longitudinal strain, early LV dilatation, impaired mechanical synchrony, and mitral regurgitation severity, have been associated with a higher PVC burden and a greater likelihood of developing PVC-CMP [1,6,15]. Moreover, improvement of these parameters after PVC suppression is a key marker of reversibility and helps distinguish true PVC-mediated dysfunction from cardiomyopathy unmasked by ectopy [15].

CMR remains the gold standard for non-invasive myocardial tissue characterization in patients with suspected PVC-CMP, as it may identify structural abnormalities in a subset of patients with significant ventricular arrhythmias despite completely normal TTE findings [31,32]. The presence, extent, and distribution of LGE provide critical prognostic information: even limited areas of LGE reflect irreversible fibrosis and are associated with a lower likelihood or slower rate of LV functional recovery after suppression of ectopy, indicating that the observed dysfunction likely represents secondary or concomitant cardiomyopathy rather than a purely arrhythmia-mediated process. Quantitative analyses have shown that a higher LGE burden or its mid-wall or subepicardial localization correlates strongly with incomplete or delayed LVEF recovery. Conversely, the absence of LGE reliably identifies patients with a high probability of full functional normalization after successful PVC suppression or ablation [31].

A complex relationship between myocardial scar, PVC site of origin, and post-ablation outcomes is particularly evident in patients with chronic ischemic cardiomyopathy, as shown by recent CMR-guided evidence: a minority of PVCs originate directly from infarct scar, while many arise from non-scarred regions (e.g., the LVOT and papillary muscles) [33]. These observations indicate that, beyond focal replacement fibrosis, chronic pressure and volume overload may lead to diffuse interstitial fibrosis in regions exposed to increased wall stress, thereby shaping the arrhythmogenic substrate and partly explaining heterogeneous ablation responses and functional recovery despite similar scar burden [16,22,33].

Advanced imaging parameters, such as echographic strain, native T1 mapping, and extracellular volume quantification, improve sensitivity for detecting this early or diffuse interstitial fibrosis and may also help anticipate the potential for reversibility. However, their prognostic value remains less firmly established and is currently considered complementary rather than definitive in clinical decision-making. Therefore, LGE detection and quantification by CMR remain the cornerstone for refining diagnosis, prognostic stratification, and guiding therapy in PVC-CMP [31].

An ischemic assessment, most commonly performed with cardiac computed tomography, is generally recommended to rule out alternative causes of ventricular dysfunction or significant ectopy. Invasive coronary angiography is reserved for patients with a significant cardiovascular risk profile and suspicion of coronary artery disease as the underlying cause of ectopy [6,7,31].

### 5.3. Surface ECG Localization Versus Non-Invasive Mapping

Careful 12-lead ECG analysis is fundamental for identifying the PVC origin and guiding the ablation strategy. Key parameters, such as the precordial transition, QRS duration, notching, and lead concordance, help refine localization and procedural planning [6,15,20,34]. When integrated with imaging and electroanatomic mapping, these findings enhance precision and improve outcomes, particularly in epicardial or non-OT foci [9,10,18].

As a general principle, PVCs exhibiting an inferior axis and a LBBB morphology are suggestive of an OT origin, most commonly the RVOT [1]. Precise differentiation between RVOT and LVOT origins is clinically relevant because it determines access, mapping strategy, procedural risk, and ablation success. A precordial transition around V3-V4 favors an RVOT focus, whereas an earlier transition in V2-V3 suggests an LVOT or aortic cusp origin [1,34]. When localization remains uncertain, several ECG-based criteria have been proposed, including R-wave amplitude in lead I, the V2S-to-V3R ratio, transitional zone indices, and other measures of the shift in the precordial R-to-S transition, although none provides absolute diagnostic certainty, particularly in anatomically overlapping regions [34]. In this context, a recent study systematically compared the diagnostic performance of the main ECG algorithms, specifically in patients with a V3 transition, and demonstrated substantial variability in sensitivity and specificity. The authors further proposed a hybrid ECG plus clinical approach incorporating male sex, a history of hypertension, and age greater than 50 years as clinical markers suggestive of an LVOT origin, with a diagnostic performance comparable to ECG criteria alone for discriminating from RVOT origin. While external validation is still needed, this combined strategy appears physiologically plausible and may offer additional support in challenging cases where surface ECG findings are equivocal [35].

Conversely, a superior axis pattern generally indicates a basal or epicardial origin, particularly when accompanied by broad QRS complexes, a slurred upstroke, precordial notching, or prolonged intrinsicoid deflection, which further support an epicardial exit [10,15]. Epicardial PVCs may arise from regions such as the LV summit or the cardiac crux, where delayed initial activation and wider QRS complexes reflect slower epicardial conduction; summit foci often show delayed intrinsicoid deflection in lateral leads, while crux PVCs exhibit deep inferior S waves and basal patterns [10,15]. Simple practical clues can support suspicion of epicardial origin in general practice: a QS or very small R wave in V1, disappearance of septal R waves, and a noticeably broad QRS (>150–160 ms) increase the likelihood of an epicardial or intramural focus [10]. A later precordial transition with predominantly positive inferior forces favors posterior sites, whereas an earlier transition with deeper anterior S waves indicates anterior or anteroseptal origins [10,34]. PVCs from mid-ventricular or apical regions typically show narrower QRS complexes than those arising from OT or basal sites, particularly near the septum, and may resemble sinus rhythm [6,10].

Fascicular PVCs arise from the LV conduction system and typically show short coupling intervals and relatively narrow QRS complexes with rsR′ or qR patterns in the inferior leads, consistent with rapid Purkinje activation rather than myocardial propagation [10,15,34]. In contrast, papillary muscle PVCs often present an RBBB morphology with axis variation depending on their anterolateral or posteromedial origin, and frequently demonstrate late precordial transition or shifting QRS morphologies due to the complex three-dimensional motion of these structures [10,15,18,34]. Broader or more heterogeneous QRS complexes, particularly those with a slurred onset or delayed intrinsicoid deflection, are more suggestive of deeper intramural or myocardial wall origins [10,34]. Fragmented or notched PVC morphologies may be associated with slow or heterogeneous conduction and, in appropriate clinical settings, suggest possible intramural or fibrotic substrates. However, this finding is nonspecific and can also be seen in structurally normal hearts, as current evidence does not establish a definitive link with myocardial fibrosis or predict reversibility after ectopy suppression [10,18,22].

Traditional localization rules remain highly reliable for common OT PVCs, where electrophysiologic studies confirm ECG predictions in approximately 70–90% of cases, but are less accurate for intramural, papillary muscle, LV summit, or crux foci due to complex geometry and epicardial fat attenuation [10,18,20,34]. Emerging non-invasive ECGi and computational mapping approaches have improved localization in these challenging contexts, reducing procedural time and fluoroscopy exposure [12]. Moreover, AI and machine-learning algorithms trained on large ECG datasets are showing increasing sensitivity for identifying complex origins, providing a valuable adjunct to conventional ECG interpretation and invasive mapping [11].

## 6. Therapeutic Strategies: Indications, Comparative Effectiveness, and Practical Selection

### 6.1. Guiding Principles and Levels of Recommendation

Management of PVCs requires balancing evidence-based guideline recommendations with individualized clinical judgment [1,2]. According to the 2022 ESC Guidelines for the Management of Ventricular Arrhythmias, catheter ablation is recommended as first-line therapy (Class I recommendation, Level of Evidence B) for symptomatic idiopathic PVCs originating from the RVOT or the left fascicles, owing to its high acute success and low complication rates [9,10]. Several contemporary reviews and expert consensus statements further support extending this indication to idiopathic PVCs arising from the LVOT, given comparable efficacy and safety profiles [10,36]. In addition, in patients with suspected PVC-CMP, catheter ablation has a similar Class I recommendation, Level of Evidence C as a strategy to reverse LV dysfunction when a causal relationship is likely [9,36]. These recommendations are supported by the ESC Guidelines and by meta-analyses and large multicenter observational series consistently demonstrating substantial improvement in LV systolic function, symptomatic relief, and durable arrhythmia suppression following successful ablation. Nevertheless, randomized trials directly comparing ablation with optimized pharmacological therapy remain scarce, and comparative estimates are largely derived from observational cohorts that cannot fully exclude selection bias or residual confounding [6,9,35,36,37,38].

A Class IIa recommendation, Level of Evidence B is assigned for catheter ablation in symptomatic idiopathic PVCs arising from sites other than the RVOT or the left fascicles, particularly when performed in experienced centers and when drug therapy is ineffective, not desired, or unavailable [9,16,17]. Nonetheless, outcomes are more heterogeneous in these locations, and long-term success is generally lower, particularly in complex substrates such as intramural or LV summit foci where accessibility is limited [10,38]. In contrast, in asymptomatic patients with a very high idiopathic PVC burden (consistently >20%), ablation may be considered with a Class IIb recommendation, Level of Evidence B, especially when there is concern for PVC-related LV remodeling or early systolic dysfunction [9,16,17]. This weaker recommendation is supported primarily by prospective and non-randomized studies demonstrating improvement or normalization of LV function after successful suppression of frequent PVCs, with consistent results across multiple cohorts. However, large randomized trials remain limited, and these data should therefore be interpreted as supportive but not definitive when weighing ablation against medical therapy in individual patients [7,36,38].

Pharmacologic therapy (Table 1) is primarily guided by symptom burden and site of origin, as outlined in the 2022 ESC Guidelines [9]. For idiopathic PVCs arising from foci other than the RVOT or the left fascicles, Class II (β-blockers) or Class IV (non-dihydropyridine calcium channel blockers) are indicated as first-line therapy (Class I recommendation, Level of Evidence C). Conversely, in symptomatic idiopathic PVCs originating from the RVOT or the left fascicles, when catheter ablation is not feasible, not desired, or has failed, Class II or Class IV agents are recommended as first-line therapy (Class IIa recommendation, Level of Evidence C), as well as Class Ic drugs in patients without structural heart disease. These recommendations are mainly supported by non-randomized studies, small prospective trials, and expert consensus. If these options prove ineffective, Class III antiarrhythmic drugs may be used selectively in highly symptomatic, refractory cases (Class IIb recommendation, Level of Evidence B) [1,7,8,9,36].

Conversely, ESC guidance assigns a Class III recommendation against the use of Class Ic agents in structural heart disease, against Class IV agents in the setting of LV dysfunction and the routine use of Class III drugs (specifically amiodarone) in asymptomatic patients and preserved ventricular function, as pharmacologic suppression in this context provides no prognostic benefit and may expose patients to unnecessary risk [1,7,9].

HF guideline-directed medical therapy, which includes angiotensin-converting enzyme inhibitors, angiotensin receptor–neprilysin inhibitors, β-blockers, mineralocorticoid receptor antagonists, and sodium–glucose cotransporter 2 inhibitors, is indicated in all cases involving LV systolic dysfunction, regardless of the underlying cause. These agents should be maintained even after recovery to enhance reverse remodeling and reduce relapse [9,29].

Alongside specific therapies, lifestyle adjustments that include reducing caffeine and alcohol intake, quitting smoking, engaging in regular physical conditioning, and managing stress may offer modest benefits [1,2,7]. However, complete resolution of ectopy is uncommon with these measures alone [1,6].

### 6.2. Pharmacologic Therapy: Efficacy, Drug Classes, and Adverse Effects

#### 6.2.1. Class I Agents

Class Ia agents, such as quinidine and hydroquinidine, may be selectively used in Purkinje-related or idiopathic PVCs, including those associated with inherited arrhythmia syndromes [1,9,20]. Their clinical use is limited by QT prolongation, gastrointestinal intolerance, and the risk of torsade de pointes [1,6]. In contemporary practice, quinidine is more commonly employed for Brugada syndrome or short QT syndrome than for isolated PVCs. However, it may also be considered in selected cases of malignant Purkinje-triggered PVCs capable of initiating ventricular fibrillation, including idiopathic forms [20,39].

Class Ib agents: Mexiletine can be used in refractory PVCs, particularly when related to post-infarction Purkinje triggers or ischemic scar tissue [1,20,39]. In small retrospective and prospective cohorts, it achieves a modest 30–40% reduction in PVC burden with acceptable tolerance. Typical adverse effects include tremor, dizziness, and gastrointestinal upset, while proarrhythmia is uncommon. However, its modest efficacy and narrow therapeutic range limit its routine use in idiopathic PVCs [1,7].

Class Ic agents, primarily flecainide and propafenone, are the most potent oral antiarrhythmics for idiopathic PVCs, particularly those arising from OT or fascicular origins [8,23]. Prospective and observational studies report that nearly complete suppression, typically defined as more than an 80% reduction in PVC burden, occurs in 40–50% of appropriately selected patients [8,36]. Their high efficacy results from potent sodium-channel blockade, which slows conduction and suppresses both reentry and triggered activity. Flecainide is generally more potent but carries a higher risk of QRS widening and conduction delay. In contrast, propafenone, due to its mild beta blocking activity, may be advantageous in adrenergically mediated PVCs but less suitable in bradycardic patients [6,8,36]. Common adverse effects include proarrhythmia, negative inotropy, and neurological symptoms such as dizziness or blurred vision [2,8,36]. Finally, although both agents are typically contraindicated in structural cardiomyopathy because of increased mortality risk demonstrated in the CAST trial, this contraindication primarily reflects “classic” scar-related heart disease [37]. Emerging observational data in arrhythmogenic cardiomyopathy suggest that flecainide may be used under specialist supervision to significantly reduce ventricular ectopy, with reported benefit in patients with and without LV involvement [40].

#### 6.2.2. Class II Agents

β-blockers (e.g., metoprolol, bisoprolol, carvedilol) are considered first-line therapy for symptomatic PVCs or catecholamine-sensitive OT foci, as they reduce sympathetic activation and stabilize cellular automaticity [1,7,9,19]. In observational and prospective cohort studies, β-blockers achieve a 20–40% mean reduction in PVC burden, providing consistent symptomatic relief, though rarely resulting in complete suppression [1,6,8]. They are particularly suitable when HF therapy is indicated, or LV dysfunction coexists [6,7].

Their efficacy is generally greater in adrenergically mediated or stress-induced PVCs, but limited in purely idiopathic forms unrelated to sympathetic tone [1,8,19]. In such cases, they may still serve as adjunctive therapy before considering catheter ablation or Class Ic agents [6,7,9]. Common adverse effects include bradycardia, hypotension, fatigue, and, less frequently, bronchospasm [1,2].

#### 6.2.3. Class III Agents

Sotalol, a Class III antiarrhythmic with additional β-blocking properties, reduces PVC burden by 30–60% in observational and registry studies. It may be particularly useful for OT or papillary-muscle PVCs, where adrenergic modulation contributes to arrhythmogenesis [8]. However, its use requires careful ECG monitoring because of the risk of QT prolongation and torsade de pointes, particularly in the presence of renal dysfunction, electrolyte imbalance, or female sex, which increase susceptibility [1,9]. For these reasons, inpatient initiation or close outpatient follow-up is recommended when starting or up-titrating therapy [1,9].

Amiodarone remains the most potent pharmacologic suppressor of PVCs, achieving approximately a 70% reduction or even complete elimination, in non-randomized and registry studies [3,36]. Its benefit extends to patients with structural heart disease in whom other antiarrhythmic drugs are contraindicated or ineffective. Because it blocks multiple channels by inhibiting sodium, potassium, and calcium currents and exerts non-competitive β-blocking effects, amiodarone effectively reduces ventricular ectopy and thereby may support partial LV functional recovery in PVC-CMP. However, its long-term use is limited by extracardiac toxicity, including thyroid, hepatic, pulmonary, and dermatologic complications, as well as numerous drug interactions, which together require close clinical and biochemical surveillance [6,7,9]. Dronedarone, a non-iodinated analog of amiodarone, was developed to reduce systemic toxicity but demonstrates limited efficacy against PVCs and is contraindicated in HF or LV dysfunction, restricting its use to isolated or anecdotal cases [1,9].

#### 6.2.4. Class IV Agents

Non-dihydropyridine calcium-channel blockers (CCBs), such as verapamil and diltiazem, have shown benefit in verapamil-sensitive fascicular PVCs or peri-Hisian origins, as they suppress triggered activity mediated by calcium-dependent afterdepolarizations [2,10,19]. In small prospective series and case-based reports, these agents typically achieve a 20–30% reduction in PVC frequency, accompanied by meaningful symptomatic improvement. In fascicular PVCs, a favorable acute response to verapamil may also help confirm the diagnosis of this subtype [10,18]. They are preferred when structural heart disease is present and Class I agents are contraindicated. Their main limitations are negative inotropy, atrioventricular block, and hypotension, which subsequently restrict use in patients with LV dysfunction [1,9].

### 6.3. Catheter Ablation: Techniques, Efficacy, and When to Avoid Intervention

**Figure 2:**
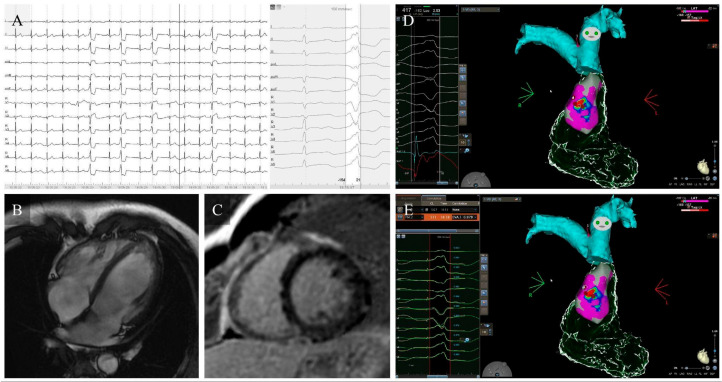
Anterior RVOT ectopic focus ablation in PVC-CMP. (**A**) Twelve-lead ECG shows frequent monomorphic PVCs (32% burden) with LBBB morphology, inferior axis, and early transition (V3), consistent with an anterior RVOT origin. (**B**,**C**) CMR demonstrates mild ventricular dilation, moderately reduced LVEF, and no LGE, supporting PVC-CMP. (**D**) High-density RVOT mapping identifies the earliest activation at the subpulmonic anterior region (25 ms preactivation) with high pace-map match. (**E**) RF ablation achieves immediate and sustained PVC suppression with subsequent normalization of LV size and LVEF.

**Figure 3:**
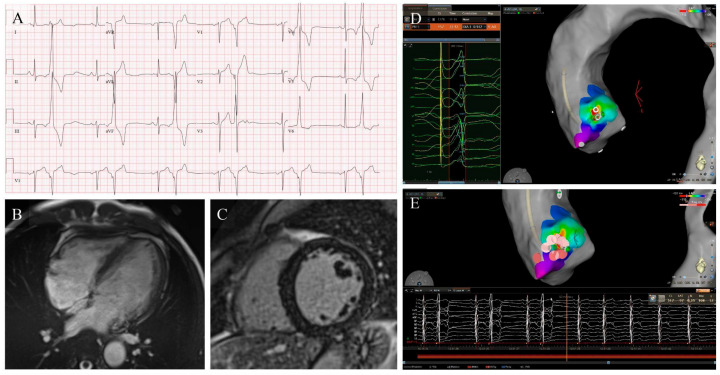
Intercoronary cusp LVOT ectopic focus ablation in PVC-CMP. (**A**) Frequent monomorphic PVCs (29% burden) with inferior axis and transition at V3; surface ECG criteria were inconclusive for RVOT versus LVOT origin. (**B**,**C**) CMR shows mild LV dilatation and systolic dysfunction without LGE, consistent with PVC-CMP. (**D**) Mapping shifts from RVOT to retroaortic LVOT, identifying earlier activation at the intercoronary cusp with high pace-map match. (**E**) RF ablation achieves immediate and sustained elimination of PVCs, with subsequent normalization of LV size and LVEF on follow-up.

**Figure 4:**
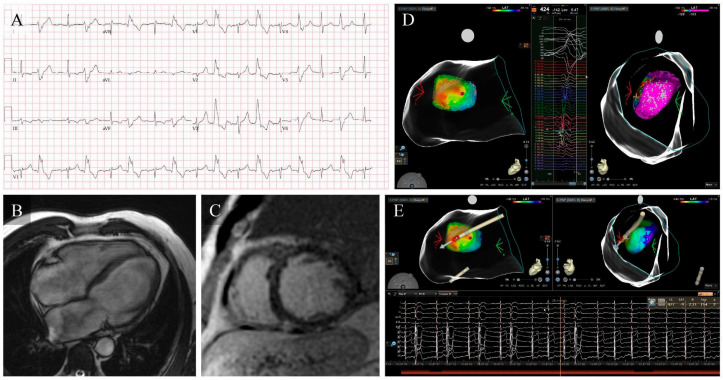
PPM ectopic focus ablation in PVC-CMP. (**A**) Baseline ECG showing frequent (44% burden) monomorphic PVCs with a wide QRS, RBBB-like morphology, inferior axis, and late precordial transition, suggestive of a PPM origin. (**B**,**C**) CMR demonstrating severe biventricular dilation and systolic dysfunction with linear mid-myocardial LGE in basal and mid anteroseptal segments. (**D**) High-density LV electroanatomical mapping identified the earliest activation at the PPM, with significant preactivation and high pace-map correlation. (**E**) RF ablation acutely suppresses PVCs, with subsequent improvement in LVEF, supporting a reversible PVC-mediated component.

Although catheter ablation is the most effective and durable therapy for eliminating focal idiopathic PVCs (Table 2) and for reversing PVC-CMP when the arrhythmic focus is accessible, its limitations in daily practice should be explicitly acknowledged, particularly clinically relevant recurrences after initially successful procedures and the still imperfect criteria for selecting patients most likely to derive prognostic and functional benefit from early ablation [6,9,36]. In large multicenter cohorts and systematic reviews, predominantly involving idiopathic PVCs arising from the RVOT (Figure 2), LVOT (Figure 3) and fascicular system, acute procedural success typically ranges from 80–95% and long-term PVC freedom from 70–85% in experienced centers [38]. These outcomes are lower for PVCs arising from more complex sites such as the PPM (Figure 4), the valvular annuli, or the LV summit (with recurrence rates reported up to 30–40%) [10,23]. Major complications remain uncommon (1–2%), consisting mainly of vascular injury, cardiac tamponade, coronary or valvular damage, and auriculoventricular block during septal or para-Hisian ablations [9,38].

Irrigated-tip RF ablation remains the standard modality due to its ability to create deep, controllable lesions and its versatility for endocardial targets, whether approached via retrograde aortic or transseptal access. Cryoablation may be advantageous near the conduction system or when catheter stability is essential [10]. Conversely, bipolar ablation, intramural needle ablation, and selective ethanol infusion through coronary venous or arterial branches offer solutions for deep-seated or refractory intramural and epicardial PVCs, particularly those within the LV summit [10,41].

Mapping accuracy is a major determinant of procedural success. Activation mapping during spontaneous PVCs remains the ideal approach, complemented by pace-mapping and QRS morphology matching when ectopy is intermittent. The use of high-density mapping systems, contact-force sensing, and intracardiac echocardiography enhances both precision and safety [10]. Notably, the absence of spontaneous PVCs during the procedure represents a relevant limitation, thus warranting minimal sedation strategies, isoproterenol infusion, or repeated mapping once clinical ectopy recurs. In a recent multicenter study of ablation for infrequent PVCs, 11% of cases lacked clinical ectopy and required exclusive pace-mapping for localization. Predictors of very low intraprocedural PVC density (<1 PVC/min) included the absence of structural heart disease, higher baseline LVEF, lower ambulatory PVC burden, and a RVOT origin. In these cases, a systematic pace-mapping protocol is feasible and effective even when only a single clinical PVC is observed during monitoring, provided that morphology matches prior ECG documentation [42]. Furthermore, recently developed non-invasive or computational mapping approaches may provide a complementary option when spontaneous ectopy is absent [10,11,12,23].

When technical or substrate-related limitations reduce the likelihood of procedural success, ablation may be deferred, particularly in patients with relevant comorbidities, multifocal or deep intramural PVCs, challenging anatomy, or active myocardial disease requiring primary treatment [7,8,9,10,14]. In such situations, careful shared decision-making is essential to balance expected benefit with procedural risk and to incorporate patient priorities [9,14]. Pharmacologic therapy becomes a reasonable alternative or a bridge when ablation is not feasible or not desired [8,9,36]. In contrast, simple observation is reserved for individuals with minimal symptoms and low PVC burden once structural disease has been excluded [2,6,8,10].

## 7. Prognosis, Long-Term Outcomes, and Follow-Up

Successful suppression of frequent PVCs, specifically by catheter ablation, is consistently associated with symptomatic relief, reversal of remodeling, and recovery of LV function. Studies report normalization of ejection fraction in 40–70% of cases, with a mean increase of 10–20% within 3–6 months after suppression [6,25,36,38]. However, in a multicenter cohort of idiopathic PVC-CMP successfully eliminated, despite two-thirds achieving an EF improvement of ≥10%, about 15% showed no recovery at all [6,25].

Although establishing a definitive survival benefit remains difficult given the predominance of observational data, preventing progression to irreversible cardiomyopathy is consistently linked to better long-term outcomes and fewer HF events [6,7,25,36]. Effective ectopy suppression further translates into improved exercise capacity and quality of life, particularly in symptomatic individuals [6,7,25].

As previously discussed, early intervention, narrower QRS duration, and absence of LGE on CMR predict faster and more complete recovery, whereas epicardial, intramural, or multifocal origins and residual fibrosis are associated with partial or delayed improvement [18,25,36]. The presence of underlying cardiomyopathy or extensive myocardial disease further limits the likelihood of full normalization, even when ectopy is successfully abolished [6,7,18]. Once diffuse interstitial fibrosis develops, a threshold of non-reversibility may be reached. Experimental data indicate that chronic ectopy can promote irreversible remodeling through progressive scarring, often undetectable by conventional imaging. Advanced modalities, such as T1 mapping and extracellular volume quantification, enhance early detection of diffuse fibrosis and may help identify this transition, supporting earlier therapeutic intervention, even in asymptomatic patients [6,7,31,36].

Follow-up of patients with PVC-CMP should include repeat ambulatory monitoring (Holter or event recorder) and imaging at 3–6 months post-therapy, with periodic reassessment to ensure sustained PVC reduction and stable LV recovery [1,6,36]. Device-based data and remote monitoring offer valuable long-term surveillance, particularly in CRT recipients, confirming pacing adequacy and enabling early detection of recurrence [28,29].

## 8. Future Directions, Evidence Gaps, and Role of AI

### 8.1. Major Gaps in Evidence

Despite major advances in understanding PVC-CMP, significant evidence gaps remain. Most data come from retrospective or single-center studies, and randomized trials directly comparing early ablation with optimized medical therapy are still scarce, leaving the optimal timing and patient selection uncertain [6,8,9,10].

Predictors of reversibility are also incompletely characterized. While imaging markers such as LGE and myocardial strain provide prognostic value, standardized thresholds and quantification protocols remain lacking across centers [6,8,31,36]. Similarly, biomarkers including NT-proBNP, galectin-3, and high-sensitivity troponins correlate with myocardial stress, neurohormonal activation, and adverse remodeling; yet their cutoff values and predictive accuracy for post-suppression recovery remain unvalidated in prospective cohorts [6,8]. In parallel, genetic studies have identified desmosomal and cytoskeletal variants that may modulate susceptibility to PVC-related LV dysfunction, though their clinical utility and integration into predictive models warrant further investigation [24,25].

Recent observational work also indicates that PVC location may independently influence long-term outcomes, including the likelihood of developing HF, beyond conventional risk factors. Thus, integrating PVC origin into future predictive models could refine risk stratification and support earlier intervention in selected patients [18]. Finally, long-term outcome data are limited, particularly regarding sustained LV recovery, recurrence, and mortality beyond mid-term follow-up. Cost effectiveness and quality of life comparisons between ablation and medical therapy are likewise scarce, and the heterogeneity in outcome definitions, including terms like “successful suppression” or “PVC-CMP remission”, highlights the need for standardized endpoints and multicenter collaboration [6,7,9,36].

### 8.2. Role of AI and Machine Learning

Machine learning and AI are rapidly transforming the management of cardiac arrhythmias. Validated applications include AI-enhanced ECG algorithms that can improve arrhythmia detection and assist PVC localization, and early clinical studies suggest that AI-supported mapping workflows may reduce procedure time and fluoroscopy use in selected ablation settings [11,12,13]. Experimental approaches include integrating AI with non-invasive computational mapping (ECGi), which is under active evaluation to improve localization performance and procedural planning across broader populations [11,12]. More hypothetical directions involve multimodal predictive models combining imaging, genetic, and continuous monitoring data to estimate reversibility and recurrence risk; these remain largely developmental and require prospective validation before routine clinical use. Collectively, these technologies may contribute to more personalized diagnosis, risk stratification, and therapeutic decision-making in patients with PVCs and related cardiomyopathies. Still, their clinical impact will depend on robust external validation and standardized implementation [25,26,27].

### 8.3. Future Research Priorities

Meaningful progress in this field will depend on the standardization of diagnostic, imaging, biomarker, and genetic criteria, as well as on the definition of uniform follow-up endpoints to enable robust comparisons and advance toward precision-guided management of PVC-CMP [10,24,25]. Prospective registries should leverage tools with existing clinical feasibility data (wearable monitoring and AI-supported mapping) to assess reproducibility and cost-effectiveness, while separately benchmarking more investigational approaches (AI-integrated ECGi and multimodal prediction models) before they inform routine care [11,12,29]. Future research priorities include randomized controlled trials comparing early ablation versus medical therapy in high-burden patients, validation of imaging and biomarker panels to predict recovery, and systematic evaluation of advanced ablation strategies for PVCs originating from complex sites such as intramural or LV summit foci [10,38,41].

## 9. Practical Conclusions and Recommendations

PVC-CMP exemplifies how a frequent and often underestimated arrhythmia can progressively remodel the ventricle and yet remain fully reversible when detected and treated early. Recognition of its reversible nature highlights the importance of systematic PVC quantification and comprehensive imaging to differentiate true PVC-mediated dysfunction from secondary cardiomyopathies. The presence or absence of myocardial fibrosis, best characterized by CMR, remains the strongest determinant of reversibility.

Therapeutic decisions should emphasize the timely suppression of ectopy. Catheter ablation provides the highest likelihood of durable rhythm control and ventricular recovery, while pharmacologic therapy remains a valuable option in selected contexts. Beyond symptom relief, early intervention prevents progression toward irreversible remodeling and HF. Management should increasingly incorporate individualized factors, including comorbidities, genetic background, and imaging markers, to guide prognosis and therapy. Follow-up should confirm reduction in PVC burden and sustained ventricular recovery through periodic monitoring and imaging.

Looking forward, bridging existing evidence gaps in long-term outcomes, genetic risk, and biomarker validation will be essential. The integration of AI and advanced mapping technologies promises to enhance diagnostic precision and procedural success. Ultimately, PVC-CMP highlights the expanding potential of precision medicine to shift arrhythmia care from reactive management toward proactive, mechanism-guided intervention.

## Figures and Tables

**Table 1:** Pharmacologic therapy for PVCs.

Drug Class and Subtypes	Targeted Ion Channel	Estimated Efficacy (and Study Type)	Main Indications by PVC Subtype/Origin	Adverse Effects & Main Limitations	Principal Contraindications	Key References
**Class Ia** **(quinidine,** **hydroquinidine)**	Na^+^ channel block, some K^+^ channel block	Modest PVC suppression (limited data, small observational & syndrome-specific cohorts)	Purkinje-related PVCs, inherited arrhythmia syndromes, malignant Purkinje-triggered PVCs capable of initiating VF	QT prolongation, torsade de pointes, GI intolerance	Long QT, significant conduction disease	[1,7,39]
**Class Ib** **(mexiletine)**	Na^+^ channel block (fast kinetics)	30–40% PVC reduction (retrospective & small prospective cohorts)	Post-infarction Purkinje triggers, ischemic-scar related PVCs	Tremor, dizziness, GI intolerance, narrow therapeutic range	Severe hepatic dysfunction	[1,4,21,39]
**Class Ic** **(flecainide,** **propafenone)**	Potent Na^+^ channel block (slow kinetics)	40–50% near-complete PVC suppression (observational & prospective cohorts)	Idiopathic **outflow-tract PVCs**, fascicular PVCs; adrenergic PVCs (propafenone beneficial due to mild β-blocking activity)	Proarrhythmia, negative inotropy, QRS widening, neurologic symptoms	Structural or ischemic heart disease (CAST)	[3,6,23,36,37]
**Class II** **(β-blockers)**	β1-adrenergic blockade	20–40% PVC reduction (observational & prospective cohorts)	Catecholamine-sensitive PVCs, PVC-CMP with **LV dysfunction**, non-ablated QT/fascicular PVCs	Bradycardia, hypotension, bronchospasm	Severe bradycardia, high-degree AV block, acute decompensated HF	[1,3,4,6,19]
**Class III** **(sotalol)**	K^+^ channel block + β-blockade	30–60% PVC reduction (observational & registry studies)	Outflow-tract PVCs, papillary-muscle PVCs	QT prolongation & torsade de pointes, requires ECG monitoring	QT prolongation, severe renal dysfunction	[1,6,7,23,38]
**Class III** **(amiodarone)**	Multi-channel block (Na^+^, K^+^, Ca^2+^) + noncompetitive β-block	≥70% PVC suppression/elimination (non-randomized & registry data)	**Structural heart disease**, contraindication to class I drugs & refractory PVC-CMP	Thyroid, pulmonary, hepatic, dermatologic toxicity, drug interactions	Severe hepatic disease, thyroid dysfunction, pulmonary fibrosis	[3,4,7,39]
**Class IV** **(verapamil, diltiazem)**	Ca^2+^ channel block	20–30% PVC reduction (small prospective & case series)	Peri-Hisian & fascicular PVCs (diagnostic value)	Negative inotropy, AV block, hypotension	Severe LV dysfunction, advanced AV block	[2,8,23]

**Table 2:** Estimated prevalence of PVCs by site of origin, acute and long-term success, specific complications, and recommended ablation techniques, noting that reported success and complication rates may vary significantly with operator experience and center volume.

PVC Site of Origin	Estimated Prevalence (%)	Acute Ablation Success (%)	Long-Term PVC Freedom (%)	Most Frequent Cardiac Specific Complications (Estimated Probability)	Recommended Techniques for Optimal Results	Key References
RVOT	~60–70%	~85–95%	~75–90%	RV perforation, cardiac tamponade (<0.5%)	High-density activation mapping, pace mapping, long-sheath support	[17,25,38]
LVOT	~10–20%	~80–90%	~70–85%	Cusp/coronary injury (<1%), air embolism (<0.5–1%)	Coronary cusp mapping, LVOT-cusp simultaneous mapping, coronary imaging	[10,25,38]
Fascicular PVCs	~5–10%	~80–90%	~75–85%	AV block/conduction system injury (<0.5–3%)	Purkinje potential targeting, activation + pace-mapping	[19,25]
Aortic sinuses of Valsalva	~5–10%	~75–90%	~65–80%	Coronary artery injury, valve leaflet injury (<1%)	Cusp angiography, bipolar mapping, coronary assessment	[10,38]
Posterior papillary muscle	~3–5%	~70–90%	~60–70%	Papillary muscle injury, cardiac tamponade (<1%)	Intracardiac echo (ICE), catheter stabilization, pace-mapping	[10,19]
Anterior papillary muscle	~2–4%	~65–85%	~55–70%	Papillary muscle injury, steam pops (<1%)	ICE guidance, rotational mapping, sheath support	[10,19,25]
Mitral annulus	~3–5%	~70–85%	~60–75%	Valve apparatus injury (<1%), cardiac tamponade (<0.5%)	Annular rotational mapping, ICE, contact-force optimization	[10,23]
Tricuspid annulus	~2–3%	~70–85%	~60–75%	Tricuspid leaflet injury (<1%), RV wall perforation (<0.5%)	RV-inflow mapping, ICE, long-sheath support	[10,19,25]
LV summit	~3–5%	~60–80%	~50–70%	Phrenic nerve/ coronary injury (<1%)	Coronary venous or aortic cusp mapping, needle or bipolar RF	[10,40]
Intramural PVCs	~2–5%	~50–70%	~40–60%	Deep myocardial injury, cardiac tamponade (<1%), steam pops (1–3%)	Needle or bipolar RF, deep-lesion irrigated systems	[10,33,40]
Epicardial idiopathic PVCs	~1–3%	~60–80%	~50–70%	Pericardial bleeding, coronary injury (<1–2%), phrenic nerve injury (1–3%)	Epicardial mapping, coronary angiography, pericardial access	[10,33,38]
Para-Hisian/ Septal OT	~1–3%	~70–85%	~60–75%	AV block (<1–2%), septal perforation (<1%)	Cryoablation, low-power RF, detailed His mapping	[1,10]
Moderator band	~1–3%	~70–85%	~60–70%	RV perforation, arrhythmia induction (<1%)	ICE, steerable sheath stabilization, pace-mapping	[19,25]
RV free wall	~1–2%	~65–85%	~55–75%	RV perforation, cardiac tamponade (<1%)	ICE, substrate mapping	[10,19,25]
Multifocal PVCs	~5–10%	~60–75%	~50–65%	Incomplete PVC suppression/ablation inefficacy (30–40%)	Priority-based morphology, mapping, sequential ablation	[6,23]

## Data Availability

No new data were generated or analyzed in this study.

## Abbreviations

The following abbreviations are used in this manuscript:

AI	Artificial intelligence
AV	Atrioventricular
CCBs	Calcium channel blockers
CMR	Cardiac magnetic resonance
CRT	Cardiac resynchronization therapy
ECG	Electrocardiogram
ECGi	Electrocardiographic imaging
ESC	European Society of Cardiology
HF	Heart failure
ICD	Implantable cardioverter defibrillator
ILR	Implantable loop recorder
LBBB	Left bundle branch block
LGE	Late gadolinium enhancement
LV	Left ventricle
LVEF	Left ventricular ejection fraction
LVOT	Left ventricular outflow tract
OT	Outflow tract
PVC	Premature ventricular complex
PVC-CMP	Premature ventricular complex-induced cardiomyopathy
PPM	Posteromedial papillary muscle
RF	Radiofrequency
RVOT	Right ventricular outflow tract
